# Biodistribution and SPECT Imaging Study of ^99m^Tc Labeling NGR Peptide in Nude Mice Bearing Human HepG2 Hepatoma

**DOI:** 10.1155/2014/618096

**Published:** 2014-05-19

**Authors:** Wenhui Ma, Zhe Wang, Weidong Yang, Xiaowei Ma, Fei Kang, Jing Wang

**Affiliations:** Department of Nuclear Medicine, Xijing Hospital, Fourth Military Medical University, No. 127 West Changle Road, Xi'an, Shaanxi Province 710032, China

## Abstract

A peptide containing Asn-Gly-Arg(NGR) sequence was synthesized and directly labeled with ^99m^Tc. Its radiochemical characteristics, biodistribution, and SPECT imaging were evaluated in nude mice bearing human HepG2 hepatoma. Nude mice bearing HepG2 were randomly divided into 5 groups with 5 mice in each group and injected with ~7.4 MBq ^99m^Tc-NGR. The SPECT images were acquired in 1, 4, 8, and 12 h postinjection via caudal vein. The metabolism of tracers was determined in major organs at different time points, which demonstrated rapid, significant tumor uptake and slow tumor washout. The control group mice were blocked by coinjecting unlabelled NGR (20 mg/kg). Tumor uptake was (2.52 ± 0.83%) ID/g at 1 h, with the highest uptake of (3.26 ± 0.63%) ID/g at 8 h. In comparison, the uptake of the blocked control group was (1.65 ± 0.61%) ID/g at 1 h after injection. The SPECT static images and the tumor/muscle (T/NT) value were obtained. The highest T/NT value was 7.58 ± 1.92 at 8 h. The xenografted tumor became visible at 1 h and the clearest image of the tumor was observed at 8 h. In conclusion, ^99m^Tc-NGR can be efficiently prepared and it exhibited good properties for the potential SPECT imaging agent of tumor.

## 1. Introduction

Angiogenic tumor vessels are important element for tumor growth and metastasis and the metalloexopeptidase CD13/aminopeptidase N (APN) plays a critical role in cancer angiogenesis. Peptides containing NGR have shown high efficiency in targeted cells, tissues, and new vessels with CD13 receptor overexpression [1, 2]. Moreover, in tissues that undergo angiogenesis, blood vessels also overexpress APN and proliferation of endothelial cells is well known to be an important factor in tumor angiogenesis [3, 4]. It is proved that NGR can bind with new vasculature by aminopeptidase N (CD13) and integrin *α*v*β*3, although the binding mechanisms are different. Meanwhile, CD13 receptor mediated binding to tumor vasculature is specific but not to the other CD13 rich tissues, which was proved by in vivo studies [5]. Good blood clearance was thought to favor the utilization of imaging techniques. Therefore, NGR peptide was characterized as a promising molecular imaging candidate for early diagnosis, particularly for tumor.

Many peptides containing NGR motif have been produced with excellent tumor targeted efficacy, such as tTF-NGR, NGR-hTNF, and cyclic NGR-labeled paramagnetic quantum dots (cNGR-pQDs) [6–10]. Our previous work indicated that both NGR monomer and dimer showed relatively high tumor uptake [5, 11].

In this study, a new NGR peptide was synthesized and labeled with ^99m^Tc, then subjected to SPECT imaging of CD13 expression in a subcutaneous mouse HepG2 hepatoma xenograft model, which was proved to show positive CD13 receptor and easy formation of tumor.

## 2. Materials and Methods

### 2.1. General

All chemicals (reagent grade) were obtained from commercial suppliers and used without further purification. NGR (YGGCNGRC) was prepared by SPPS using the Fmoc method on a chlorotrityl chloride resin. ^99m^TcO_4_^−^ was produced from ^99^Mo/^99m^Tc generator (Beijing Atom High Tech, China). Water was purified using a Milli-Q ultrapure water system from Millipore (Milford, USA), followed by passing through a Chelex 100 resin before bioconjugation and radiolabeling. Radio-TLC was performed on silica gel-coated plastic sheets (Polygram SIL G, Macherey-Nagel) with acetone and Vethanol : Vammonia water : Vwater = 2 : 1 : 5 as the eluents. The plates were read with Bioscan Mini-scan (USA) and Allchhrom Plus software. The semipreparative high-performance liquid chromatography (HPLC, Aglint, Canada) was employed for peptide analysis. NGR-containing peptide was prepared by solid phase peptide synthesis (SPPS) using the Fmoc strategy on chlorotrityl chloride resins as previously reported [12]. Mass spectra were used to confirm the identity of the products. Mass spectra were obtained on a Q-Tof premier-UPLC system equipped with an electrospray interface (ESI; Waters, USA) or a Thermo Electron Finnigan LTQ mass spectrometer equipped with an electrospray ionization source (Thermo Scientific, USA).

### 2.2. Radiolabelling and Formulation

The fresh ^99m^TcO_4_^−^ solution (37–74 MBq) was added into a solution of NGR peptide (15~20 *μ*g peptide per mCi ^99m^TcO_4_^−^) with 200 *μ*g stannous chloride dissolved in 1 M HCl (5 *μ*g/*μ*L) and 20 *μ*L of 0.2 M NaAc/HAc buffer (pH = 4.2) solution. The mixture was incubated at room temperature for 30 min. The ^99m^TcO_4_^−^-containing solution was filtered over a 0.2 *μ*m syringe filter (Acrodisc, PALL, USA) and then passed through a 0.2 *μ*m Millipore filter into a sterile vial for use.

### 2.3. In Vitro Stability

The stability of ^99m^Tc-labeled NGR peptide in PBS and mouse serum at 37°C was studied at 1, 3, 6, and 12 h. Then the percentage of parent tracer was determined by radio-TLC (Table 1).

### 2.4. Cell Culture and Animal Model

HepG2 cells were grown in high glucose DMEM culture medium. All cell lines were cultured in medium supplemented with 10% (v/v) fetal bovine serum (Gibco, USA), 1% mycillin, and 1% Glutamine (Beyotime, China) at 37°C in a humidified atmosphere with 5% CO_2_. Using female BALB/c nude mice (4–6 weeks of age), HepG2 tumor model was established by subcutaneous injection of 2 × 10^6^ HepG2 tumor cells (0.1 mL) into the right upper flanks. When the tumor volume reached 0.8~1.0 cm in diameter (2-3 weeks after inoculation), the tumor-bearing mice were used for SPECT imaging and biodistribution studies. All animal studies were approved by Clinical Center at the FMMU.

### 2.5. Cell Uptake Study

HepG2 cells were seeded into 48-well plates at a density of 2 × 10^5^ cells per well 24 h prior to the experiment. HepG2 cells were then incubated with ^99m^Tc-labeled NGR peptides (~370 kBq/well) at 37°C for 15, 30, 60, 120, and 240 min. After incubation, tumor cells were washed three times with ice cold PBS and harvested by trypsinization with 0.25% trypsin/0.02% EDTA (Hyclone, USA). Cell suspensions were collected and measured in a gamma counter (Zhida, Shannxi, China). Cell uptake data was presented as percentage of total input radioactivity added to the culture medium after decay correction. Experiments were performed twice with triplicate wells.

### 2.6. Cell Binding Assay

In vitro CD13 receptor binding affinity and specificity of ^99m^Tc-NGR were assessed via competitive cell binding assay. The best-fit 50% inhibitory concentration (IC_50_) values for the HepG2 cells were calculated by fitting the data with nonlinear regression using Graph-Pad Prism5.0 (Graph-Pad Software, San Diego, CA, USA).

### 2.7. SPECT Imaging

HepG2 tumor-bearing animals were imaged in supine position with a one-head SPECT MPR (GE, USA) equipped with a pinhole collimator. About 7.4 MBq of ^99m^Tc-labeled NGR peptide was intravenously injected into each mouse under intraperitoneal injection of sodium pentobarbital at a dose of 45.0 mg/kg. Static SPECT images were acquired at 1, 4, 8, and 12 h pi. The acquisition count limit was set at 200 k.

### 2.8. Biodistribution Studies

Nude mice bearing human HepG2 hepatoma were randomly divided into 5 groups and injected with ~7.4 MBq of ^99m^Tc-NGR with or without excess unlabelled NGR peptide (20 mg/kg). After injection of the tracer, mice were sacrificed and dissected. The radioactivity in the HepG2 tumor, major organs, and muscle were collected and weighed wet with tubes (%ID/g). Mean uptake (%ID/g) for a group of animals was calculated with standard deviations. Values were expressed as mean ± SD (*n* = 5/group).

## 3. Results

### 3.1. Chemistry and Radiochemistry

**Figure 1:**
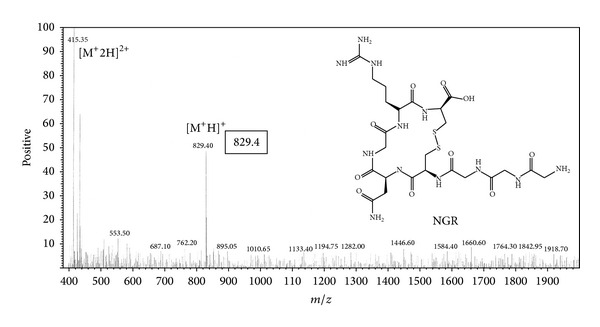
Chemical structure and mass result of NGR (YGGCNGRC).

**Figure 2:**
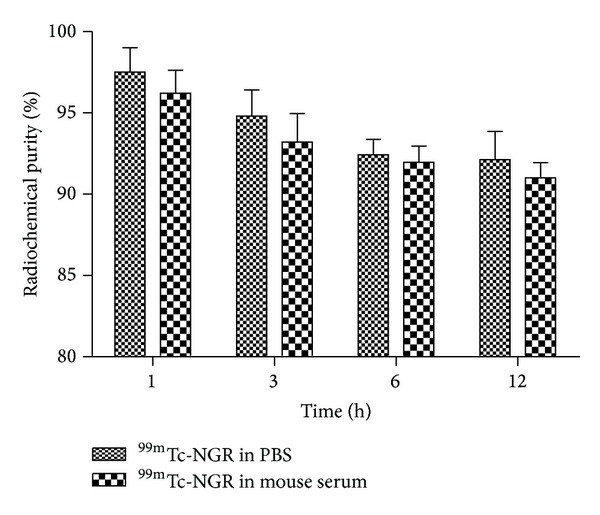
Stability of ^99m^Tc-NGR peptide in PBS (pH = 7.4) and mouse serum at 37°C for 1, 3, 6, and 12 h. Its radiochemical purity was >98%. 92% of ^99m^Tc-NGR peptide almost remained intact in PBS and mouse serum after 12 h of incubation.

NGR peptide was well prepared (Figure 1). The analytical HPLC and mass spectroscopy were used to confirm the identity of the products. The mass spectroscopy data and chemical structures for NGR were represented below (Figure 1). The electrospray ionization mass spectra of NGR were determined to be *m*/*z* = 829.40 ([M+H]^+^). After purification, the specific activity of ^99m^Tc-labeled tracers was determined to be about 3.08~6.17 MBq/nmol. The labeling yield of the product was 95 ± 0.35% and the radiochemical purity was greater than 98%. The in vitro stability of ^99m^Tc-NGR in PBS (pH 7.4) at 37°C was shown in Figure 2. After 12 h of incubation, more than 92% of ^99m^Tc-NGR peptide remained intact in mice serum.

### 3.2. Cell Uptake

**Figure 3:**
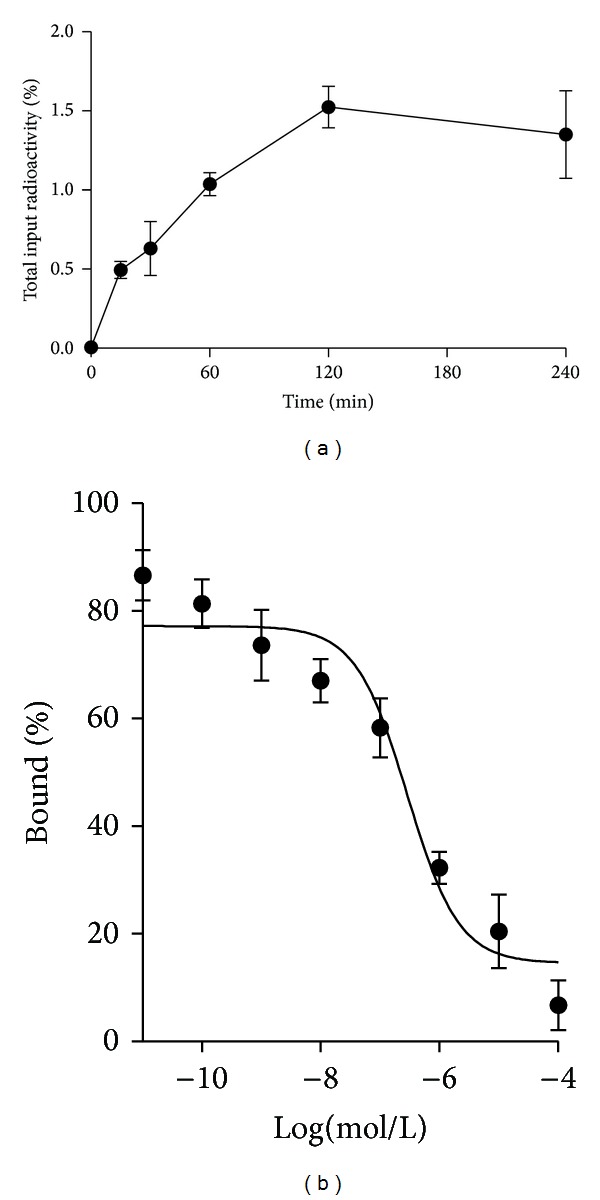
In vitro cell uptake assay and cell-binding assay of HepG2 human hepatoma cells. (a) Cell uptake assay of ^99m^Tc-NGR (*n* = 3, mean ± SD). The background readings are reflected at time 0. (b) Cell binding assay of ^99m^Tc-NGR on CD13 receptor of HepG2 cells (*n* = 3, mean ± SD).

Cell uptake study revealed that ^99m^Tc-NGR bound to HepG2 tumor cells directly. During the first 15 min, about 0.49 ± 0.05% of ^99m^Tc-NGR uptake in HepG2 cells were determined. After 2 h incubation, the peptide uptake in HepG2 cells reached the maximum of 1.52 ± 0.13% (Figure 3(a)). About 1.35 ± 0.27% of ^99m^Tc-NGR were still associated with HepG2 cells after 4 h incubation.

### 3.3. Cell Binding Assay

Ligand-receptor binding affinities of ^99m^Tc-NGR to CD13 were determined by a competitive cell-binding assay. ^99m^Tc-NGR inhibited the binding of NGR peptide to HepG2 cells in a concentration-dependent manner (Figure 3(b)). The IC_50_ values for ^99m^Tc-NGR were calculated to be 287 ± 34 nmol/L.

### 3.4. SPECT Imaging

**Figure 4:**
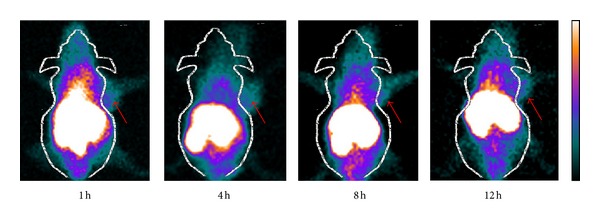
Representative decay-corrected whole-body SPECT images of mice bearing HepG2 tumors on right front flank after intravenous administration of ^99m^Tc-NGR (~7.4 MBq) SPECT images of nude mice bearing HepG2 tumor at 1, 4, 8, and 12 h pi (tumors are indicated by red arrows).

The tumor-targeting efficacy of ^99m^Tc-NGR probe in HepG2 tumor-bearing nude mice was evaluated by static SPECT scans at different time points after injection. Representative decay-corrected images are shown in Figure 4. The HepG2 tumors were clearly visualized with good tumor-to-background contrast for the tracer. Overall, ^99m^Tc-NGR provided better image quality with the same amount of injected activity.

### 3.5. Biodistribution Studies

**Figure 5:**
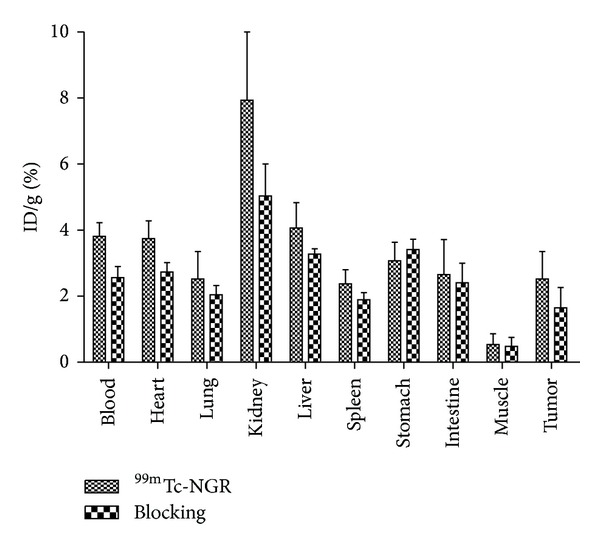
Biodistribution of ^99m^Tc-NGR (~7.4 MBq) in athymic nude mice bearing HepG2 tumor with or without NGR (20 mg/kg) at 1 h (*n* = 5, mean ± SD).

Tissue distribution data for ^99m^Tc-NGR in mice bearing HepG2 hepatoma tumors are given as percentage administered activity per gram of tissue (%ID/g) in Table 2 and Figure 5. The in vivo biodistribution of with and without coinjection of nonradiolabeled NGR peptide (20 mg/kg of mouse body weight) was examined in HepG2 tumor-bearing mice. For ^99m^Tc-NGR, the tumor uptake was determined to be 2.52 ± 0.83, 3.03 ± 0.71, 3.26 ± 0.63, and 2.81 ± 0.25% ID/g at 1, 4, 8, and 12 h, respectively. ^99m^Tc-NGR exhibited 7.93 ± 2.13% ID/g kidney uptake and 4.07 ± 0.76% ID/g liver uptake at 1 h pi. The nonspecific uptake in the muscle was at a very low level. ^99m^Tc-NGR exhibited high tumor uptake at the early time point (Figure 5), indicating the specific binding and relatively longer circulation time.

A decrease of radioactivity was observed in all dissected tissues and organs similar to SPECT imaging results in blocking group (Table 2), with the change of tumor uptake being the most significant reducing markedly from 2.52 ± 0.83% ID/g whereas the presence of nonlabeled NGR peptide significantly reduced to 1.65 ± 0.61% ID/g at 1 h after injection. For ^99m^Tc-NGR nonblocking group, 4.07 ± 0.76% ID/g in liver and 7.93 ± 2.13% ID/g in kidney were decreased to 3.27 ± 0.16% ID/g and 5.03 ± 0.97% ID/g by blocking, respectively.

## 4. Discussion

In this study, we synthesized a novel NGR peptide and investigated its biological targeting specificity, which turned out to be a promising tumor molecular imaging probe for clinical practice.

^99m^Tc has favorable chemical and physical properties and can be produced from the generator directly [11]. CD13 receptor is an attractive biological target, which has been found to be overexpressed on newly formed neovasculature and on a wide range of tumor cells types. The high radiochemical purity of the radiotracer (>98%) stimulated further analysis encompassing in vitro and in vivo evaluation, without the time consuming steps of purification and drying of the compound. The ^99m^Tc-labeled tracer also showed good stability (Figure 2) and affinity (Figure 3). The results showed that imaging acquisition after injection within 12 h is enough for detecting tumor clearly. The labeling process in this study is so convenient that the probe is practical in future clinical imaging.

The development of radiolabeled peptides for diagnostic and therapeutic applications has expanded exponentially in the last decades [8, 13, 14]. Peptide-based radiopharmaceuticals can be produced easily and inexpensively and have many favorable properties, including fast clearance, rapid tissue penetration, and low antigenicity [6, 9, 15–17]. In this study, the cysteine beside NGR motif formed a cyclic via a disulfide linkage and the direct labeling method resulted in a very stable product. At the meantime, breaking the disulfide linkage in the NGR-containing peptide during the directly labeling process may explain the slightly lower binding affinity compared with our previous results [18]. Otherwise, the extra three glycines were added to protect the core motif NGR and may increase peptide half-life and stability [19]. Meanwhile, the liver and kidney uptake was obviously reduced compared with previous study, which may also be caused by adding glycine [18].

SPECT scans of ^99m^Tc-NGR in nude mice bearing HepG2 hepatoma showed notable uptake in tumor and dominant renal and hepatic clearance. But the unspecific binding on the other tissues was decreasing and the tumor to nontumor ratio was consequently increasing. The receptor specificity of ^99m^Tc-NGR was further confirmed by effective inhibition of tumor uptake in the presence of excess nonlabeled NGR peptide in biodistribution study (Table 1). Although the ^99m^Tc-NGR uptake in liver and gastrointestinal tract was lower than previous results, the practice in detecting tumor and metastases in the abdominal area is inapplicable. Since the excretion of the probe was mainly renal, fast blood depuration should be another favorable feature.

In brief, our data demonstrated that synthesizing novel ^99m^Tc-NGR was a promising synthetic strategy for SPECT imaging in terms of in vitro and in vivo properties. Our future work will continually focus on more optimal approach to reduce liver and gastrointestinal tract uptake by modifying the peptide structure and keep the specificity binding. Additionally, a thorough comparison between various NGR peptides is warranted to screen the better radiotracers.

## 5. Conclusion

NGR peptide was successfully labeled with the generator-produced ^99m^Tc for SPECT imaging of tumor CD13 receptor. ^99m^Tc-NGR exhibited good properties in terms of binding affinity, cellular uptake, tumor uptake and retention, and pharmacokinetics. ^99m^Tc-NGR peptide is a potential SPECT agent for imaging and early diagnosis of tumor.

## Figures and Tables

**Table 1:** Rf values of free ^99m^TcO_4_^−^, ^99m^Tc-colloid and ^99m^Tc-NGR.

Rf value	Free ^99m^TcO_4_^−^	^ 99m^Tc-colloid	^ 99m^Tc-NGR
Acetone	0.9~1.0	0.0	0.0
Vethanol : Vammonia water : Vwater = 2 : 1 : 5	0.9~1.0	0.0	0.7~0.8

**Table 2:** Biodistribution data (%ID/g) of ^99m^Tc-NGR in HepG2 hepatoma tumor-bearing nude mice at 1, 4, 8, and 12 h postinjection (*n* = 5, mean ± SD).

	1 h	4 h	8 h	12 h	Blocking (1 h)
Blood	3.82 ± 0.41	1.75 ± 0.39	0.98 ± 0.22	0.75 ± 0.31	2.56 ± 0.34
Heart	3.74 ± 0.54	2.26 ± 0.33	1.15 ± 0.32	1.07 ± 0.25	2.73 ± 0.29
Lung	2.52 ± 0.83	2.13 ± 0.44	1.86 ± 0.38	1.21 ± 0.31	2.04 ± 0.28
Liver	4.07 ± 0.76	3.85 ± 0.73	3.44 ± 0.52	3.26 ± 0.47	3.27 ± 0.16
Kidney	7.93 ± 2.13	7.03 ± 0.95	6.21 ± 0.43	5.96 ± 0.41	5.03 ± 0.97
Spleen	2.37 ± 0.43	2.09 ± 0.60	1.88 ± 0.42	1.72 ± 0.37	1.89 ± 0.22
Stomach	3.07 ± 0.56	2.20 ± 0.26	1.61 ± 0.39	1.56 ± 0.32	3.41 ± 0.31
Intestine	2.65 ± 1.06	2.18 ± 0.96	1.93 ± 0.22	1.60 ± 0.27	2.40 ± 0.59
Muscle	0.53 ± 0.33	0.46 ± 0.11	0.43 ± 0.14	0.39 ± 0.22	0.48 ± 0.27
Tumor	2.52 ± 0.83	3.03 ± 0.71	3.26 ± 0.63	2.81 ± 0.25	1.65 ± 0.61

Tumor-to-normal tissue uptake ratio at 1 h postinjection					

			T/M	4.75 ± 0.91	
			T/L	0.62 ± 0.33	
			T/K	0.32 ± 0.15	

## References

[1] Wang X, Wang Y, Chen X, Wang J, Zhang X, Zhang Q (2009). NGR-modified micelles enhance their interaction with CD13-overexpressing tumor and endothelial cells. *Journal of Controlled Release*.

[2] Zhao B-J, Ke X-Y, Huang Y (2011). The antiangiogenic efficacy of NGR-modified PEG-DSPE micelles containing paclitaxel (NGR-M-PTX) for the treatment of glioma in rats. *Journal of Drug Targeting*.

[3] Bhagwat SV, Lahdenranta J, Giordano R, Arap W, Pasqualini R, Shapiro LH (2001). CD13/APN is activated by angiogenic signals and is essential for capillary tube formation. *Blood*.

[4] Negussie AH, Miller JL, Reddy G, Drake SK, Wood BJ, Dreher MR (2010). Synthesis and in vitro evaluation of cyclic NGR peptide targeted thermally sensitive liposome. *Journal of Controlled Release*.

[5] Chen K, Ma W, Li G (2013). Synthesis and evaluation of ^64^Cu-labeled monomeric and dimeric NGR peptides for MicroPET imaging of CD13 receptor expression. *Molecular Pharmacology*.

[6] Arap W, Pasqualini R, Ruoslahti E (1998). Cancer treatment by targeted drug delivery to tumor vasculature in a mouse model. *Science*.

[7] Curnis F, Arrigoni G, Sacchi A (2002). Differential binding of drugs containing the NGR motif to CD13 isoforms in tumor vessels, epithelia, and myeloid cells. *Cancer Research*.

[8] Dijkgraaf I, Yim C-B, Franssen GM (2011). PET imaging of *α*v*β*3 integrin expression in tumours with68Ga-labelled mono-, di- and tetrameric RGD peptides. *European Journal of Nuclear Medicine and Molecular Imaging*.

[9] Meng J, Yan Z, Xue X (2007). High-yield expression, purification and characterization of tumor-targeted IFN-*α*2a. *Cytotherapy*.

[10] Yang Y-S, Zhang X, Xiong Z, Chen X (2006). Comparative in vitro and in vivo evaluation of two ^64^Cu-labeled bombesin analogs in a mouse model of human prostate adenocarcinoma. *Nuclear Medicine and Biology*.

[11] Wang Z, Ma W, Wang J (2013). Imaging and therapy of hSSTR2-transfected tumors using radiolabeled somatostatin analogs. *Tumor Biology*.

[12] Adar L, Shamay Y, Journo G, David A (2011). Pro-apoptotic peptide-polymer conjugates to induce mitochondrial-dependent cell death. *Polymers for Advanced Technologies*.

[13] Chen K, Sun X, Niu G (2012). Evaluation of ^64^Cu labeled GX1: a phage display peptide probe for PET imaging of tumor vasculature. *Molecular Imaging and Biology*.

[14] Wu Y, Zhang X, Xiong Z (2005). microPET imaging of glioma integrin *α*v*β*3 expression using ^64^Cu-labeled tetrameric RGD peptide. *Journal of Nuclear Medicine*.

[15] Ndinguri MW, Solipuram R, Gambrell RP, Aggarwal S, Hammer RP (2009). Peptide targeting of platinum anti-cancer drugs. *Bioconjugate Chemistry*.

[16] Corti A, Giovannini M, Belli C, Villa E (2010). Immunomodulatory agents with antivascular activity in the treatment of non-small cell lung cancer: focus on TLR9 agonists, IMiDs and NGR-TNF. *Journal of Oncology*.

[17] Chen K, Conti PS (2010). Target-specific delivery of peptide-based probes for PET imaging. *Advanced Drug Delivery Reviews*.

[18] Ma W, Kang F, Wang Z (2013). ^99m^Tc-labeled monomeric and dimeric NGR peptides for SPECT imaging of CD13 receptor in tumor-bearing mice. *Amino Acids*.

[19] Samli KN, McGuire MJ, Newgard CB, Johnston SA, Brown KC (2005). Peptide-mediated targeting of the islets of Langerhans. *Diabetes*.

